# Antiretroviral Activity, Pharmacokinetics, and Safety of MK-8527, an Oral Nucleoside Reverse Transcriptase Translocation Inhibitor, in Adults With HIV-1 Who Had Not Previously Taken Antiretroviral Agents: Results From 2 Open-Label, Phase 1 Studies

**DOI:** 10.1093/cid/ciag135

**Published:** 2026-03-14

**Authors:** Russ P Carstens, Yash Kapoor, Ryan C Vargo, Arinjita Bhattacharyya, Graigory Garrett, Caroline Cilissen, Adedayo Adedoyin, Xiaowei Zang, Jean-Francois Denef, Carlien Leyssens, Tom Reynders, Liliana Preotescu, Richard Kaplan, Mohammed Rassool, Johannes Lombaard, Anca Streinu-Cercel, Randolph P Matthews, S Aubrey Stoch, Marian Iwamoto, Gillian L Gillespie

**Affiliations:** MRL, Merck & Co., Inc., Rahway, New Jersey, USA; MRL, Merck & Co., Inc., Rahway, New Jersey, USA; MRL, Merck & Co., Inc., Rahway, New Jersey, USA; MRL, Merck & Co., Inc., Rahway, New Jersey, USA; MRL, Merck & Co., Inc., Rahway, New Jersey, USA; MRL, MSD Belgium, Brussels, Belgium; MRL, Merck & Co., Inc., Rahway, New Jersey, USA; MRL, Merck & Co., Inc., Rahway, New Jersey, USA; MRL, MSD Belgium, Brussels, Belgium; MRL, MSD Belgium, Brussels, Belgium; MRL, MSD Belgium, Brussels, Belgium; Department of Internal Medicine, National Institute for Infectious Diseases “Prof. Dr. Matei Bals,” Bucharest, Romania; LGBT+ Health Division/GSH Trials Unit, Desmond Tutu Health Foundation, Cape Town, South Africa; Clinical HIV Research Unit, University of Witwatersrand, Helen Joseph Hospital, Johannesburg, South Africa; Research and Development, Josha Research, Bloemfontein, South Africa; Department of Internal Medicine, National Institute for Infectious Diseases “Prof. Dr. Matei Bals,” Bucharest, Romania; Infectious Diseases Department, Carol Davila University of Medicine and Pharmacy, Bucharest, Romania; MRL, Merck & Co., Inc., Rahway, New Jersey, USA; MRL, Merck & Co., Inc., Rahway, New Jersey, USA; MRL, Merck & Co., Inc., Rahway, New Jersey, USA; MRL, Merck & Co., Inc., Rahway, New Jersey, USA

**Keywords:** HIV-1, antiretroviral, nucleoside reverse transcriptase translocation inhibitor, pharmacokinetics, safety

## Abstract

**Background:**

MK-8527 is a novel oral nucleoside reverse transcriptase translocation inhibitor under clinical development as HIV-1 (HIV) prevention. Two Phase 1 single-dose monotherapy studies were conducted to evaluate antiretroviral activity, pharmacokinetics (PK), and safety of MK-8527 in adults with HIV who had not previously taken antiretroviral agents.

**Methods:**

In 2 Phase 1 studies, participants received a single oral dose of MK-8527 (0.25, 0.5, 1, 3, or 10 mg). Reduction in viral load (measured as log_10_ plasma HIV RNA copies/mL at 7 days post-dose), PK of plasma MK-8527 through 7 days, intracellular MK-8527-triphosphate (TP, the active form of MK-8527) through 28 days, exposure–response relationship, and safety through 28 days were assessed. Adverse events were descriptively summarized.

**Results:**

In total, 37 participants completed the studies. After single doses of MK-8527 0.5 to 10 mg, the mean decrease in HIV RNA at 7 days-post dose was ≥1.0 log_10_ copies/mL. The inhibitory quotient (defined as the ratio of MK-8527-TP concentration at 168 hours post-dose [C_168_] to the mean intracellular concentration of MK-8527-TP at the half-maximal inhibitory concentration [IC_50_]) exceeded 3 at single doses of ≥0.5 mg. MK-8527 at all dose levels was well tolerated, with a limited number of mild or moderate adverse events that were determined by investigators to be unrelated to the study treatment.

**Conclusions:**

In adults with HIV who had not previously taken antiretroviral agents, single doses of MK-8527 as low as 0.5 mg achieved ≥1.0 log_10_ decreases in HIV RNA at 7 days post-dose administration.

**Clinical Trials Registration:**

www.clinicaltrials.gov NCT03615183, NCT05494736

## Teaser

Two Phase 1 studies evaluated single doses of MK-8527, a nucleoside reverse transcriptase translocation inhibitor, in adults with previously untreated HIV-1. Single doses of MK-8527 ≥ 0.5 mg achieved ≥1.0 log_10_ decreases in HIV-1 RNA 7 days after dose administration.

HIV-1 (referenced as HIV hereafter) and HIV-2 remain significant global health challenges. In 2023, ∼40 million people were diagnosed with HIV-1 and HIV-2 globally, more than a million people had new HIV-1 and HIV-2 acquisitions, and ∼630 000 people died of HIV-1 and HIV-2– related disease [1]. Despite a global reduction of 39% in HIV-1 and HIV-2 acquisitions since 2010 and a 51% decrease in the number of HIV-1 and HIV-2-related deaths, some regions, including Central and Eastern Europe, Central Asia, North Africa, and the Middle East, have experienced increasing HIV-1 and HIV-2 incidence and mortality rates, highlighting the continued need for preventive and therapeutic advances to reduce transmission, morbidity, and mortality [1, 2].

Antiretroviral drugs are effective at preventing HIV acquisition when taken as pre-exposure prophylaxis (PrEP) [3–6]. Currently, 2 daily oral regimens, 1 vaginal ring, and 2 long-acting injectables are approved as PrEP. Clinical studies confirm that tenofovir disoproxil fumarate (TDF) alone or in combination with emtricitabine (FTC) is effective for PrEP [3, 4]. Daily oral tenofovir alafenamide (TAF) plus FTC has emerged as another efficacious option for PrEP [5]; however, due to limited efficacy and safety data, FTC/TAF is not indicated for individuals who may be exposed to HIV via receptive vaginal sex [7]. Pre-exposure prophylaxis effectiveness is highly dependent on adherence [8, 9], which is suboptimal in some populations at increased likelihood of HIV exposure, such as adolescents, women in sub-Saharan Africa, and people who inject drugs. Common reasons for poor adherence include social stigma, an unacceptable dosing regimen, and side effects [6, 9, 10]. Long-acting drugs provide additional prophylactic options. In 2021, cabotegravir, an intramuscular injectable given every 8 weeks, was approved for PrEP after being highly effective at preventing HIV acquisition [11–13]. In 2025, the twice-yearly subcutaneous injection, lenacapavir, was approved for PrEP following Phase 3 studies showing that it was well tolerated and highly efficacious [14, 15]. Acceptance and accessibility of long-acting injectables are limited by health care service delivery constraints, frequent clinic visits, and potential injection-site reactions [16, 17]. Therefore, more accessible, better tolerated, and more convenient antiretroviral regimens are still needed to overcome adherence barriers and improve overall treatment outcomes. A long-acting oral PrEP option could benefit people for whom access limitations and concerns about injection-site reactions may reduce adherence to injections [18].

MK-8527 is an oral nucleoside reverse transcriptase translocation inhibitor (NRTTI) in development as once-monthly PrEP that shows potent inhibition of HIV replication in human peripheral blood mononuclear cells (PBMCs) [19]. MK-8527 is phosphorylated intracellularly to its active triphosphate (TP) form, which inhibits HIV reverse transcriptase via 2 mechanisms: immediate translocation inhibition and delayed chain termination [19]. Preclinical studies show that MK-8527 is highly potent and has pharmacokinetics (PK) that support long-acting oral dosing. The PK profile of MK-8527-TP is similar to that of the active phosphorylated form of islatravir, another investigational NRTTI [20–22]. Although decreases in total lymphocyte and CD4+ T-cell counts were previously observed with higher doses of islatravir [23–25], the lower doses currently in clinical development have not demonstrated decreases in lymphocytes or CD4+ T-cell counts. Decreases in lymphocyte and CD4+ T-cell counts have not been observed with any administered doses of MK-8527. In vitro studies in human PBMCs showed that MK-8527 has subnanomolar potency (half-maximal inhibitory concentration [IC_50_] = 9.2 fmol/10^6^ cells) against HIV [19]. No off-target activities were found when MK-8527 or MK-8527-TP was tested in a panel of >100 enzyme and receptor binding assays [19].

In 2 Phase 1 clinical studies in adults without HIV, single (0.5–200 mg) and multiple (5–40 mg once-weekly for 3 weeks) doses of MK-8527 were generally well tolerated [26]. After single dosing, MK-8527 was rapidly absorbed [26]. Plasma MK-8527 concentrations decreased in a biphasic manner, with an apparent terminal half-life (t_1/2_) of 6–62 hours [26]. Plasma exposure of MK-8527 was approximately dose proportional across the dose range studied [26]. The maximum concentration (C_max_) of MK-8527-TP was reached at a median of 12–48 hours, with a t_1/2_ of 94–266 hours across doses [26]. No clinically meaningful effect of food on the PK of MK-8527 was observed [26]. We report the main findings for 2 single-dose, monotherapy Phase 1 studies evaluating the antiretroviral activity, PK, and safety of MK-8527 in adults with HIV who had not previously taken antiretroviral agents.

## METHODS

### Study Design

P002 (NCT03615183, protocol MK-8527-002, single site in Romania) and P004 (NCT05494736, protocol MK-8527-004, multiple sites in Romania and South Africa) were open-label, single-dose, monotherapy studies of MK-8527 in adults with HIV who had not previously taken antiretrovirals. Participants were enrolled into 1 of 3 panels per study (5–8 participants each) and received a single oral dose of MK-8527 (0.25–10 mg; Supplementary Figure 1). Antiviral activity, PK, and safety/tolerability were assessed at the previous dose for ≥168 hours (7 days, P002) or 240 hours (10 days, P004), to inform dose selection for subsequent panels. All doses of MK-8527 were administered after an 8-hour fast. Participants were encouraged, but not required, to initiate antiretroviral therapy ≤30 days after administration of MK-8527 (or ∼5×t_1/2_ of MK-8527-TP in PBMCs); the exact timing and regimen were decided by the participant in consultation with their physician, to decrease the likelihood of developing HIV resistance mutations [27]. In P002, viral load and PK data were collected for 10 days after MK-8527 dosing. In P004, viral load and PK data were collected for 10–28 days after MK-8527 administration. After Day 10, participants were given the option of initiating antiretroviral therapy. Safety was monitored for up to 28 days after MK-8527 dosing.

Both studies were conducted in accordance with local or national regulations (including all application data protection laws and regulations), the International Conference on Harmonisation Good Clinical Practice guidelines, and the ethical principles originating from the Declaration of Helsinki regarding independent ethics committee review, informed consent, and the protection of human participants in biomedical research. Both studies were approved by the appropriate institutional review boards and regulatory agencies.

### Participants

Participants were adults aged 18–60 years with HIV, who had not previously taken antiretroviral agents. In P002, this was defined as no prior investigational antiretroviral use for >30 consecutive days and no combination antiretroviral therapy for >60 consecutive days. In P004, this was defined as no prior marketed antiretroviral agent use; investigational antiretroviral agents for treatment or PrEP were permitted if administered ≥30 days before study treatment. HIV status was determined by a positive enzyme-linked immunosorbent assay or quantitative polymerase chain reaction. Participants with HIV RNA ≥5000 copies/mL within 30 days before study treatment and a CD4+ T-cell count >200 cells/mm^3^ at screening were eligible for inclusion. Before the administration of MK-8527, participants underwent HIV resistance testing. Participants identified with common nucleos(t)ide reverse transcriptase inhibitor resistance mutations (including, but not limited to, M184 V/I) were excluded. Male participants were required to be abstinent or use contraception. Female participants were required to be neither pregnant nor breastfeeding and to either be of non-childbearing potential, abstinent, or using a highly effective method of contraception. Participants were excluded if their estimated creatine clearance was ≤90 mL/min based on the Cockcroft-Gault equation (P002) or estimated glomerular filtration rate was ≤80 mL/min/1.73 m^2^ based on the 2021 Chronic Kidney Disease Epidemiology Collaboration Creatinine equation (P004). Participants were excluded if they were positive for the hepatitis B virus surface antigen or had a history of chronic hepatitis C virus infection and if they were unable to refrain from the use of any medication.

### Assessments and Analysis

#### Antiretroviral Activity

Blood samples were collected from the participants at multiple time points throughout the study to assess viral load (pre-dose and at 4, 12, 24, 96, 120, 144, 168, 192, 240 hours post-dose). For participants for whom viral load was assessed beyond 10 days, blood collections also occurred at 336 (Day 15), 504 (Day 22), and 672 hours (Day 29). The viral load data from all participants, measured as plasma HIV RNA log_10_ copies/mL, were pooled across doses and analyzed using a longitudinal data analysis model with fixed effects for dose, time (pre-dose, 168 hours post-dose), and dose-by-time interaction, and a random effect for participant. The change from baseline at 168 hours (7 days) was generated for each dose. Both studies included a 1 mg dosing panel; therefore, for this dose, the mean difference in the change from baseline was calculated for each study separately and both studies together.

The primary hypothesis of these studies was that a single dose of MK-8527 would achieve a mean reduction in HIV RNA ≥1.0 log_10_ copies/mL from baseline after 7 days. A ≥70% posterior probability for ≥1 well-tolerated dose level would support this hypothesis. With 6 participants in each dosing panel, there was ∼80% power to achieve ≥70% posterior probability if the true standard deviations of the log_10_ reduction from baseline in plasma HIV RNA at 168 hours post-dose were 0.3, 0.4, or 0.5 and true mean log_10_ reduction was ≥1.11, 1.12, or 1.13 log_10_, respectively.

#### Pharmacokinetics

Pharmacokinetics measurements of MK-8527 and MK-8527-TP were assessed in plasma and in PBMCs, respectively. Blood was collected for the assessment of plasma MK-8527 at pre-dose and at 0.25, 0.5, 1, 2, 3, 4, 6, 8, 12, 24, 48, 72, 96, 168 hours post-dose. Blood collection time points for the measurement of intracellular MK-8527-TP were the same as for the measurement of viral load (pre-dose and at 4, 12, 24, 96, 120, 144, 168, 192, 240 hours post-dose). For participants for whom viral load was assessed beyond 10 days, blood collections also occurred at 336, 504, and 672 hours post-dose. The PK parameters assessed for plasma MK-8527 and intracellular MK-8527-TP included area under the concentration time curve from time 0 to 168 hours post-dose (AUC_0-168_), C_max_, concentration at 168 hours post-dose (C_168_), time at maximum concentration (T_max_), and t_1/2_. Pharmacokinetics values were natural log-transformed and analyzed using a linear model with a fixed effect for dose level. Pharmacokinetics parameters were summarized for each dosing panel as least squares geometric mean and 95% CI.

#### MK-8527 Exposure–Response Relationship

The relationship between the antiviral activity of MK-8527 and the PK of MK-8527-TP was evaluated using a maximum exposure (E_max_) model to compare change from baseline in plasma HIV RNA log_10_ copies/mL and intracellular MK-8527-TP concentrations at 168 hours (7 days) after dosing for each dose level.

#### Safety

Participants were monitored throughout the study for the occurrence of adverse events (AEs) and events of clinical interest. Furthermore, a full physical examination, selected laboratory tests (hematology, serum chemistry, and urinalysis), vital sign measurements (heart rate and blood pressure), and electrocardiograms were collected prior to dose administration and at 1 hour (vital signs and electrocardiograms only) and 1, 7, and 28 days post-dosing.

## RESULTS

### Study Population

In P002, 17 participants enrolled in and completed the study between 11 February 2019 and 26 September 2019. In P004, 20 participants enrolled in and completed the study between 11 November 2022 and 31 January 2024. Participants were given a single dose of MK-8527, 10 mg (n = 6; P002), 3 mg (n = 6; P002), 1 mg (n = 13; P002/P004), 0.5 mg (n = 6; P004), and 0.25 mg (n = 6; P004) (Table 1). In P002, most participants were male (82%) and all were White (100%), whereas in P004, most participants were female (65%) and most were Black or African American (80%). The median (range) age of participants in P002 was 29 years (23–45) and 29 years (18–54) in P004. Before treatment with MK-8527, the mean (range) of plasma HIV RNA was higher in P002 than in P004, at 165 401 copies/mL (11 827–719 357) and 71 542 copies/mL (1989–253 802), respectively. In P002, one participant had HIV subtype B, and the remaining participants had subtype F. In P004, 16 participants had subtype C, 3 had subtype B, and 1 had subtype F. Viral load data were available for 18 of 20 participants enrolled in P004 14 days post-dose administration and for 10 participants both 21 days and 28 days post-dose administration. None of the participants in either study reported use of any antiretroviral agent for treatment or PrEP prior to enrollment.

**Table 1.** Disposition, Demographics, and Baseline Characteristics

Characteristic	P002			P004		
	10 mg n = 6	3 mg n = 6	1 mg n = 5	1 mg n = 8	0.5 mg n = 6	0.25 mg n = 6
Male, n (%)	4 (66.7)	5 (83.3)	5 (100)	3 (37.5)	0	4 (66.7)
Age, median (range), years	28.5 (26–32)	31.0 (23–45)	29.0 (26–39)	30.0 (19–45)	23.0 (20–28)	41.5 (18–54)
Race, White, n (%)	6 (100)	6 (100)	5 (100)	0	0	4 (66.7)
Race, Black or African American, n (%)	0	0	0	8 (100)	6 (100)	2 (33.3)
Not Hispanic or Latino, n (%)	6 (100)	6 (100)	5 (100)	8 (100)	6 (100)	6 (100)
Pre-dose plasma HIV RNA, mean (range), copies/mL	206 105 (16 787 to 719 357)	125 490 (11 827 to 408 018)	164 450 (44 671 to 264 072)	57 844 (1989 to 181 362)	77 862 (2644 to 253 802)	83 487 (24 409 to 197 067)
Pre-dose plasma HIV RNA, mean (range), log_10_ copies/mL	5.31 (4.22 to 5.86)	5.10 (4.07 to 5.61)	5.22 (4.65 to 5.42)	4.76 (3.30 to 5.26)	4.89 (3.42 to 5.40)	4.92 (4.39 to 5.29)

Abbreviation: HIV, human immunodeficiency virus.

### Antiretroviral Activity

**Figure 1.**
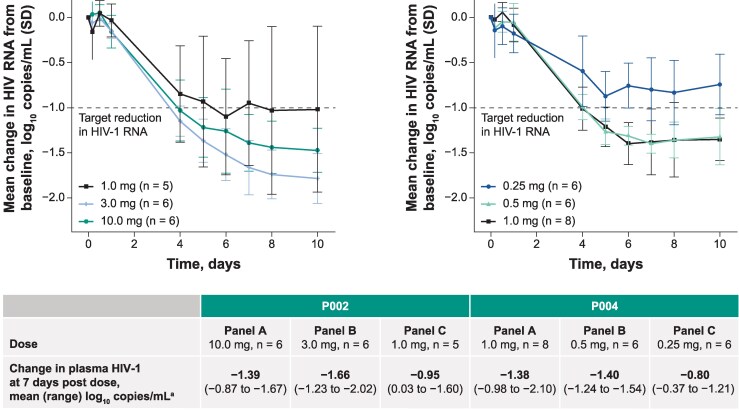
Change in HIV RNA after single doses of MK-8527. ^a^Least squares mean change from baseline. Abbreviations: HIV, human immunodeficiency virus; VL, viral load.

After a single dose of MK-8527, a mean reduction in plasma HIV RNA compared with baseline was observed for all dose levels (Figure 1). Single doses of MK-8527 0.5–10 mg achieved the ≥1.0 log_10_ copies/mL threshold for mean reduction of HIV RNA. However, the mean reduction in HIV RNA 7 days after administration of the 0.25 mg dose was below the 1.0 log_10_ copies/mL threshold (Figure 1). Combined study results for the 1 mg dose (13 participants) showed a mean reduction in plasma HIV RNA of 1.21 log_10_ copies/mL 7 days post-dose administration. Each participant treated with MK-8527 0.5–10 mg achieved a viral load reduction ≥1.0 log_10_ copies/mL 7 days after administration, with the exception of 2 participants from the 1 mg dosing panel in P002 and 1 participant from the 1 mg dosing panel in P004 (Supplementary Figure 2).

### Pharmacokinetics

After a single dose, MK-8527 was rapidly absorbed, with a median T_max_ of 0.5 hours, in plasma (Table 2). Plasma exposure (C_max_) of MK-8527 increased in an approximately dose-proportional manner. Decreases in plasma MK-8527 concentration over time followed a biphasic pattern with a geometric mean (GM) t_1/2_ of 3–40 hours at doses of 1–10 mg. Intracellular MK-8527-TP reached a C_max_ at a median time of 18–24 hours. The GM t_1/2_ of MK-8527-TP in PBMCs ranged from 80 to 211 hours.

**Table 2.** Pharmacokinetic Profile for Plasma MK-8527 and Intracellular MK-8527-TP

	PK Parameter, GM (95% CI)	P002			P004		
		10 mg n = 6	3 mg n = 6	1 mg n = 5	1 mg n = 8	0.5 mg n = 6	0.25 mg n = 6
**MK-8527**	AUC_0-168_,^a^ h·µmol/L	0.926 (0.719, 1.19)	0.152 (0.118, 0.196)	0.0189 (0.0138, 0.0258)^b^	0.0304 (0.0221, 0.0419)^c^	NR^c,d^	NR^c,d,e^
	C_max_,^a^ µmol/L	0.177 (0.114, 0.276)	0.0371 (0.0238, 0.0577)	0.0162 (0.00998, 0.0264)	0.0224 (0.0172, 0.0292)	0.00935 (0.00688, 0.0127)	0.00469 (0.00335, 0.00656)
	T_max_, hours^f^	0.50 (0.50, 1.00)	0.50 (0.50, 1.00)	0.50 (0.50, 0.50)	0.50 (0.50, 1.00)	0.50 (0.50, 3.00)	0.50 (0.23, 1.00)
	t_1/2_, hours^g^	40.31 (60.27)	12.36 (78.23)	NR	3.25 (54.52)	NR^c^	NR^c^
**MK-8527-TP**	AUC_0-168_,^a^ h·pmol/10^6^ cells	178 (140, 226)	64.1 (50.5, 81.3)	29.1 (22.4, 37.7)	29.2 (24.1, 35.3)	12.6 (10.1, 15.7)	5.53 (4.44, 6.89)
	C_max_,^a^ pmol/10^6^ cells	1.81 (1.40, 2.35)	0.644 (0.497, 0.834)	0.265 (0.200, 0.352)	0.270 (0.232, 0.314)	0.134 (0.113, 0.160)	0.0535 (0.0449, 0.0637)
	C_168_,^a^ pmol/10^6^ cells	0.509 (0.371, 0.698)	0.334 (0.244, 0.458)	0.0978 (0.0692, 0.138)	0.111 (0.0808, 0.152)	0.0484 (0.0344, 0.0681)	0.0224 (0.0154, 0.0325)
	T_max_, hours^f^	18.00 (12.00, 24.00)	24.00 (12.00, 24.00)	24.00 (12.00, 96.63)	24.00 (12.00, 24.05)	24.03 (12.07, 24.68)	23.91 (12.00, 24.12)
	t_1/2_, hours^g^	117.84 (35.00)	188.66 (122.50)	210.93 (14.62)	128.24 (49.00)	172.09 (90.23)	80.15 (52.35)

Abbreviations: AUC_0-168_, area under the concentration time curve from time 0 to 168 hours after the dose; AUC_last_, area under the concentration-time curve from the time of dosing to the time of the last measurable concentration; C_168_, concentration at 168 hours; C_max_, maximum concentration; CI, confidence interval; CV, coefficient of variation; GM, geometric mean; NR, not recorded; t_1/2_, apparent terminal half-life; PK, pharmacokinetics; T_max_, time to maximum concentration; TP, triphosphate.

^a^Back-transformed least squares mean and 95% CI from linear fixed effects model performed on natural log-transformed values.

^b^N = 4.

^c^The majority of MK-8527 plasma samples for 0.5 mg and 0.25 mg were below the lower limit of quantification. Therefore, only C_max_ and T_max_ were evaluable.

^d^In P004, this value is AUC_last_.

^e^N = 5. One participant in the 0.25 mg dosing panel had a complete plasma concentration profile below the limit of quantification and was excluded from the analysis.

^f^Median (range) reported for T_max_.

^g^GM and percentage geometric CV reported for t_1/2_.

### MK-8527 Exposure–Response Relationship

**Figure 2.**
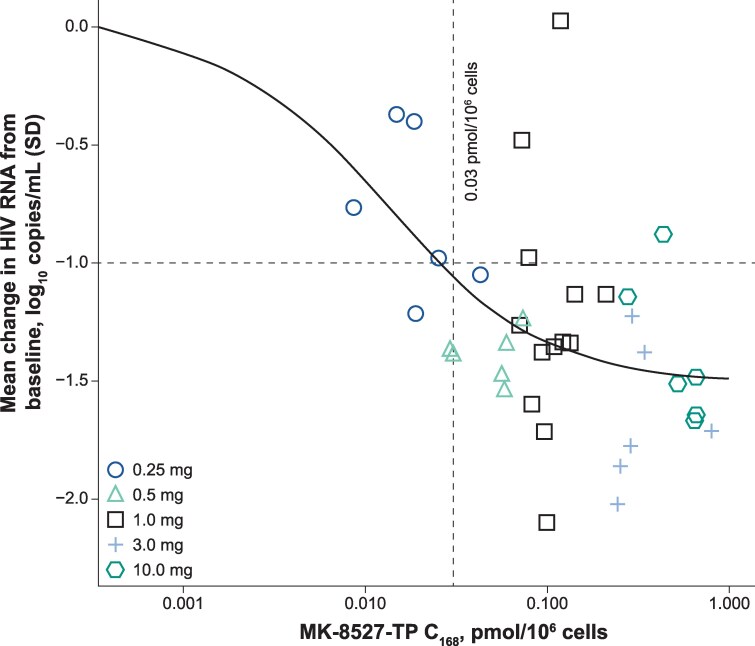
Exposure–response model for a single-dose MK-8527 (0.25–10 mg). For 1 participant in the 1 mg panel, PK sample at 168 h was not analyzed due to bioanalytical limitations, and the 168-h concentration was estimated based on the 144- and 192-h samples using linear interpolation. For 1 participant in the 0.25 mg panel, the 168-h sample was below the limit of quantification. The concentration was estimated as half of the lower limit of quantification (LLOQ/2). Abbreviations: C_168_, concentration at 168 h; HIV, human immunodeficiency virus; PK, pharmacokinetics.

An E_max_ model was used to compare reductions in plasma HIV RNA from baseline to 7 days after MK-8527 administration and intracellular MK-8527-TP concentrations at 7 days after each dose (Figure 2). Based on the model, an efficacy threshold for MK-8527-TP was set at 0.03 pmol/10^6^ PBMCs, which is conservatively expected to provide the targeted ≥1.0 log_10_ reduction in viral load in adults with HIV. MK-8527-TP concentrations above this threshold would be expected to cover wild-type HIV, but not genetic variants such as M184I/V. Using the in vitro IC_50_ for MK-8527-TP against HIV in human PBMCs (9.2 fmol/10^6^ PBMCs) [19], the inhibitory quotient (IQ [trough concentration]]/[IC_50_] in vitro) at the 0.03 pmol/10^6^ PBMCs PK efficacy target is ∼3.

### Safety

In both studies, MK-8527 was generally well tolerated (Supplementary Tables 1 and 2). In P002, 2 participants (11.8%) reported ≥1 AE. Adverse events included pharyngitis, dizziness, and insomnia. In P004, 12 participants (60.0%) reported ≥1 AE. The most common AEs (>1 participants) were headache (4 participants; 2 participants received the 1 mg dose and 1 participant each received 0.25 and 0.5 mg doses), impetigo, tinea faciei, and urinary tract infection (2 participants each; 1 participant each who received 0.5 and 1 mg doses). All AEs were mild or moderate in intensity. No serious AEs, events of clinical interest, or deaths were reported, and no participant discontinued the study due to AEs. None of the AEs in either study was determined by the investigator to be drug-related. There were no clinically meaningful changes in laboratory assessments, including total lymphocyte counts (Supplementary Tables 3 and 4), vital signs, or electrocardiogram measurements following MK-8527 administration. No cases of pregnancy were reported in either study.

## DISCUSSION

These 2 Phase 1 studies represent the first administration of single doses of MK-8527 to people with HIV. The results provide critical data on antiretroviral activity, PK, PK/pharmacodynamics, and safety of this novel NRTTI in adults with HIV and inform dose selection for HIV prevention in people who have a high likelihood of HIV exposure.

The primary hypothesis was that at single-dose levels, MK-8527 would reduce mean HIV RNA by ≥1.0 log_10_ copies/mL compared with baseline after 7 days. Single doses of MK-8527 (0.5–10 mg) achieved this threshold of antiviral activity. For 1 mg doses in P002, 1 participant out of 5 did not show a ≥1.0 log_10_ copies/mL reduction after 7 days, and 1 participant out of 5 had no antiviral response. The reason for this is unknown. In P004, 7 of 8 participants achieved this threshold after 7 days with 1 mg doses, and 1 participant had a viral load reduction of 0.98 log_10_ copies/mL after 7 days. For the 0.25 mg and 0.5 mg doses, all participants showed antiviral activity, and all participants in the 0.5 mg panel achieved a ≥1.0 log_10_ copies/mL reduction in viral load after 7 days.

The PK results showed that MK-8527 is rapidly absorbed and that the active form, MK-8527-TP, has a long t_1/2_ in PBMCs consistent with preclinical and prior Phase 1 studies [19, 26]. Utilizing data from the current studies, the efficacy threshold of MK-8527-TP was set to 0.03 pmol/10^6^ PBMCs, corresponding to an IQ of 3. Compared with IQs from clinical and preclinical data from other approved oral PrEP agents, the data suggest that MK-8527 would be effective in preventing HIV acquisition [28]. The favorable PK profile and subnanomolar potency of MK-8527-TP support further investigation of MK-8527 as a possible long-acting oral option for prevention of HIV.

These Phase 1 studies were an important part of a larger development program to support dose selection for future clinical studies. Three doses of MK-8527 (3, 6, 12 mg, administered once-monthly) were selected using population PK modeling and evaluated in a Phase 2 study of participants with a low likelihood of exposure to HIV (NCT06045507) [28, 29]. Two Phase 3 studies of once-monthly MK-8527 as PrEP, EXPrESSIVE-11 (NCT07044297) and EXPrESSIVE-10 (NCT07071623), are ongoing [30, 31].

Safety findings from these Phase 1 studies align with safety findings from previous Phase 1 studies and showed that MK-8527 was generally well tolerated, with no serious AEs reported [26].

Study limitations include the small sample size and imbalances in participant demographics and baseline viral loads across dosing panels, potentially limiting the interpretation of the results. Furthermore, any potential impact of sex, race, body mass, or other participant characteristics was not evaluated in this study. Only single-dose administration was assessed, limiting the evaluation of safety; however, viral load reduction after single-dose administration is generally indicative of viral load reduction with multiple-dose administration. Limited viral load sampling to 10 days restricts the evaluation of treatment duration, warranting further clinical assessment to understand its full effect and durability.

## CONCLUSIONS

In adults with HIV who had not previously taken antiretroviral agents, single doses of MK-8527 as low as 0.5 mg achieved ≥1.0 log_10_ decrease in HIV RNA by 7 days post-dose administration. The safety and PK profiles of MK-8527 support continued clinical investigation for use as once-monthly oral PrEP.

## Supplementary Material

ciag135_Supplementary_Data

## Supplementary Data

Supplementary materials are available at Clinical Infectious Diseases online. Consisting of data provided by the authors to benefit the reader, the posted materials are not copyedited and are the sole responsibility of the authors, so questions or comments should be addressed to the corresponding author.
