# Supplementary material for: Antiretroviral Activity, Pharmacokinetics, and Safety of MK-8527, an Oral Nucleoside Reverse Transcriptase Translocation Inhibitor, in Adults With HIV-1 Who Had Not Previously Taken Antiretroviral Agents: Results From 2 Open-Label, Phase 1 Studies
Source: Clin Infect Dis. 2026 Mar 14;82(5):e984–91. doi: 10.1093/cid/ciag135 (PMC13189651; doi:10.1093/cid/ciag135)
Supplement: ciag135_Supplementary_Data [file ciag135_supplementary_data.docx]

**Supplementary Materials**

**Supplementary Figure S1. CONSORT diagram**.

**
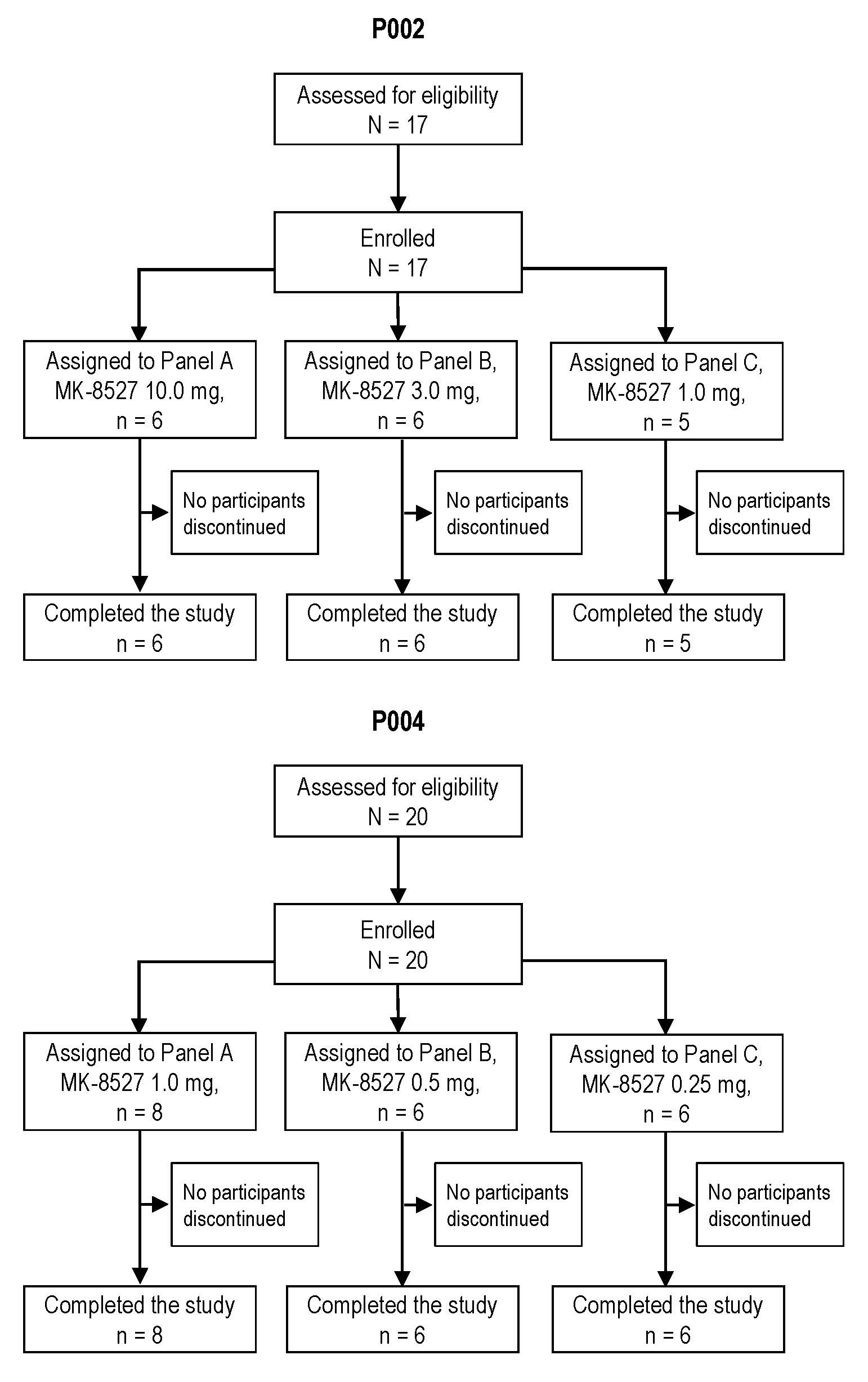
**

**Supplementary Figure S2. Summary of antiretroviral activity in individual participants**. Horizontal dotted line represents the targeted viral load reduction of −1.0 log_10_ copies/mL HIV-1 RNA.


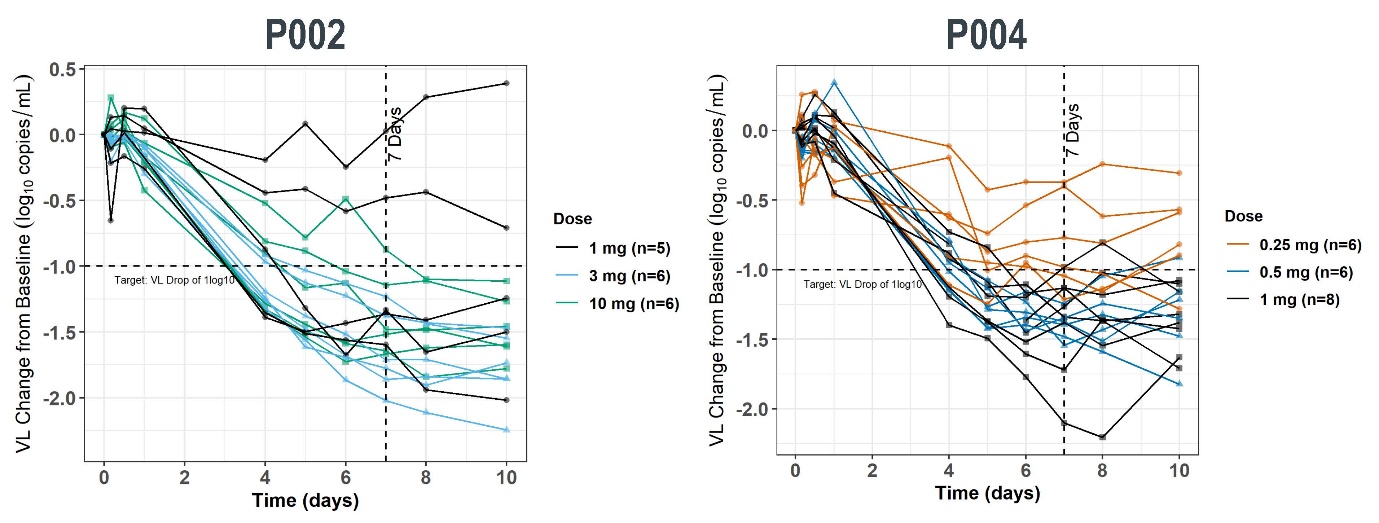


**Supplementary Table S1. Adverse event summary for P002**

| **Participants, n (%)** | **MK-8527**  **10 mg** | **MK-8527**  **3 mg** | **MK-8527**  **1 mg** | **Total** |
| --- | --- | --- | --- | --- |
| With ≥1 AEs | 0 | 2 (33) | 0 | 2 (11.8) |
| With drug-related^a^ AEs | 0 | 0 | 0 | 0 |
| With serious AEs | 0 | 0 | 0 | 0 |
| With serious drug-related^a^ AEs | 0 | 0 | 0 | 0 |
| Discontinued due to AEs | 0 | 0 | 0 | 0 |
| Discontinued due to drug-related^a^ AEs | 0 | 0 | 0 | 0 |
| Died | 0 | 0 | 0 | 0 |

Abbreviation: AE, adverse event.

^a^Considered by the investigator to be related to the study drug.

**Supplementary Table S2. Adverse event summary for P004**

| **Participants, n (%)** | **MK-8527**  **1.0 mg** | **MK-8527**  **0.5 mg** | **MK-8527**  **0.25 mg** | **Total** |
| --- | --- | --- | --- | --- |
| With ≥1 AEs | 5 (62.5) | 5 (83.3) | 2 (33.3) | 12 (60.0) |
| With drug-related^a^ AEs | 0 | 0 | 0 | 0 |
| With serious AEs | 0 | 0 | 0 | 0 |
| With serious drug-related^a^ AEs | 0 | 0 | 0 | 0 |
| Discontinued due to AEs | 0 | 0 | 0 | 0 |
| Discontinued due to drug-related^a^ AEs | 0 | 0 | 0 | 0 |
| Died | 0 | 0 | 0 | 0 |

Abbreviation: AE, adverse event.

^a^Considered by the investigator to be related to the study drug.

**Supplementary Table S3. Mean percent change from baseline in total lymphocyte counts (10^9^ cells/L) following administration of** **multiple doses of MK-8527 in P002**

|  | **Baseline^a^** | | **Poststudy visit^a^** | | **Change from baseline** | | **Percent change from baseline  LS mean^b^ (90% CI)** |
| --- | --- | --- | --- | --- | --- | --- | --- |
| **Dose** | **n** | **Mean (SD)** | **n** | **Mean (SD)** | **n** | **Mean (SD)** |  |
| **10 mg** | 6 | 1.67 (0.529) | 6 | 1.96 (0.539) | 6 | 0.287 (0.163) | 19.0 (3.98, 34.0) |
| **3 mg** | 6 | 1.88 (0.694) | 6 | 1.88 (0.608) | 6 | −0.00167 (0.348) | 2.88 (−12.1, 17.9) |
| **1 mg** | 5 | 2.43 (0.653) | 5 | 2.35 (0.396) | 5 | −0.0800 (0.664) | 1.76 (−14.7, 18.2) |

Abbreviation: LS, least squares.

^a^Arithmetic means and SDs were provided for baseline, poststudy visit, and change from baseline.

^b^The LS means and their 90% CIs for percent change from baseline were estimated from a linear mixed-effects model containing fixed effects terms for treatment, timepoint, time point, and treatment by time point interaction, with a compound symmetry covariance matrix to model the correlation between different time points within the same participant.

Baseline measurements were taken at Day 1, predose. Poststudy visit was 28 days after dose.

**Supplementary Table S4.** **Mean percent change from baseline in total lymphocyte counts (10^9^ cells/L) following administration of** **multiple doses of MK-8527 in P004**

|  | **Baseline^a^** | | **Poststudy visit^a^** | | **Change from baseline** | | **Percent change from baseline  LS mean^b^ (90% CI)** |
| --- | --- | --- | --- | --- | --- | --- | --- |
| **Dose** | **n** | **Mean (SD)** | **n** | **Mean (SD)** | **n** | **Mean (SD)** |  |
| **1.0 mg** | 8 | 1.99 (0.968) | 8 | 1.87 (0.710) | 8 | −0.120 (0.320) | 0.357 (−15.9, 16.6) |
| **0.5 mg** | 6 | 1.71 (0.423) | 6 | 1.86 (0.589) | 6 | 0.148 (0.305) | 8.16 (−10.6, 26.9) |
| **0.25 mg** | 6 | 1.66 (0.693) | 6 | 1.47 (0.165) | 6 | −0.183 (0.631) | 0.203 (−18.5, 18.9) |

Abbreviation: LS, least squares.

^a^Arithmetic means and SDs were provided for baseline, poststudy visit, and change from baseline.

^b^The LS means and their 90% CIs for percent change from baseline were estimated from a linear mixed-effects model containing fixed effects terms for treatment, timepoint, time point, and treatment by time point interaction, with a compound symmetry covariance matrix to model the correlation between different time points within the same participant.

Baseline measurements were taken at Day 1, predose. Poststudy visit was 28 days after dose.
